# Association between the apolipoprotein E gene polymorphism and ischemic stroke in Chinese populations: New data and meta-analysis

**DOI:** 10.3892/etm.2012.866

**Published:** 2012-12-19

**Authors:** LIAN GU, LI SU, QING CHEN, BAOYUN LIANG, YUWANG QIN, JUANJUAN XIE, GUANGLIANG WU, YAN YAN, JIANXIONG LONG, HUAYU WU, JINJING TAN, WEIHUA DOU, WEI CHEN, PENG WU, JINPING WANG

**Affiliations:** 1 Department of Internal Neurology, First Affiliated Hospital, Guangxi University of Chinese Medicine, Nanning, Guangxi 530023;; 2 School of Public Health, Guangxi Medical University, Nanning, Guangxi 530021;; 3 Guangxi University of Chinese Medicine, Nanning, Guangxi 530001;; 4 School of Basic Medicine, Guangxi Medical University, Nanning, Guangxi 530021, P.R. China

**Keywords:** apolipoprotein E gene, polymorphism, ischemic stroke, association study, meta-analysis

## Abstract

Ischemic stroke (IS) is a complex multifactorial inherited disease. Many studies have focused on the potential genetic effects of apolipoprotein E (ApoE) gene polymorphism on IS. However, inconsistencies still exist in the association of ApoE gene polymorphism with IS. The aim of this study was to investigate the ApoE gene polymorphism in relation to IS in the Guangxi Han populations and assess the risk of various ApoE genotypes associated with IS in Chinese populations. We conducted a case-control study involving a total of 166 IS cases and 192 healthy controls to investigate the association of ApoE gene polymorphism with IS in the Guangxi Han populations. Furthermore, we performed a meta-analysis to investigate whether the ApoE gene polymorphism is associated with IS in Chinese populations. There was no evidence for a significant association between ApoE gene polymorphism and IS in the Guangxi Han populations (ɛ2/ɛ2 vs. ɛ3/ɛ3: OR=1.25, 95% CI=0.08–20.17; ɛ2/ɛ3 vs. ɛ3/ɛ3: OR=1.49, 95% CI=0.79–2.79; ɛ2/ɛ4 vs. ɛ3/ɛ3: OR=1.25, 95% CI=0.17–9.00; ɛ3/ɛ4 vs. ɛ3/ɛ3: OR=1.10, 95% CI=0.60–2.04; ɛ4/ɛ4 vs. ɛ3/ɛ3: OR=2.50, 95% CI=0.22–27.87; allele ɛ2 vs. allele ɛ3: OR=1.39, 95% CI=0.80–2.44; allele ɛ4 vs. allele ɛ3: OR=1.16, 95% CI=0.68–1.98). In our meta-analysis, a significant association of ApoE gene polymorphism with IS was found in the genetic model of ɛ2/ɛ4 vs. ɛ3/ɛ3 (OR=2.04, 95% CI=1.45–2.85), ɛ3/ɛ4 vs. ɛ3/ɛ3 (OR=1.93, 95% CI=1.42–2.62), ɛ4/ɛ4 vs. ɛ3/ɛ3 (OR=3.41, 95% CI=2.17–5.34) and allele ɛ4 vs. allele ɛ3 (OR=2.34, 95% CI=1.91–2.86). However, no clear associations were found in the model of ɛ2/ɛ2 vs. ɛ3/ɛ3 (OR=1.56, 95% CI=0.90–2.71), ɛ2/ɛ3 vs. ɛ3/ɛ3 (OR=0.93, 95% CI=0.79–1.09) and allele ɛ2 vs. allele ɛ3 (OR=1.10, 95% CI=0.97–1.25). In conclusion, no association was found between ApoE gene polymorphism and IS in the Guangxi Han populations, while the results of the meta-analysis indicate that the ApoE mutation allele ɛ4 increases the risk of IS in Chinese populations.

## Introduction

Stroke is one of the leading causes of mortality and disability throughout the world (1). It was estimated that stroke affects 15 million people worldwide according to the World Health Organization. Five million of these patients suffer from permanent disability and approximately 5.5 million people succumbed. Over half of these mortalities were found in Asian countries, including India, Bangladesh, Pakistan, China, Japan and Korea (2). Ischemic stroke (IS) is a complex, multifactorial, inherited disease. It has a high incidence and mortality rate, as well as a high risk of reoccurrence and disability and has become a threat to health and a heavy burden for families and society. It was estimated that more than 7 million people in China have been diagnosed with cerebrovascular disease, of which 70% were IS (3). It is well accepted that genetic factors are associated with the onset of cerebrovascular diseases (4). Numerous studies have focused on the association between apolipoprotein E (ApoE) gene polymorphism and IS.

The ApoE gene, located on chromosome 19q13.2, is a candidate gene for the development of IS through its effect on lipid metabolism (5). Polymorphism of the ApoE gene was first observed by Utermann *et al* in 1975 (6). The ApoE gene is considered to possess three major alleles: ɛ2, ɛ3 and ɛ4. One allele of ApoE is inherited from each parent, therefore yielding six possible genotypes: ɛ2/ɛ2, ɛ2/ɛ3, ɛ2/ɛ4, ɛ3/ɛ3, ɛ3/ɛ4 and ɛ4/ɛ4 (7). It is well accepted that ɛ3/ɛ3 is a wild-type genotype with a frequency of approximately 67% and is the most common genotype in healthy and diseased subjects (8). The most common allele isoform is ɛ3 with a frequency of approximately 70–80%. The other two dysfunctional allele isoforms, ɛ2 and ɛ4, have a frequency of 5–10% and 10–15%, respectively (9).

A number of clinical studies have reported that the ɛ4 allele is associated with ischemic cerebrovascular disease (ICVD) (10,11). However, this is inconsistent with other studies, which showed no association between ApoE gene polymorphism and ICVD (12,13). Many association studies and meta-analysis concerning the association of ApoE gene polymorphism and IS have been conducted. However, in view of the inconsistent results among clinical studies, we conducted a case-control study to further investigate the association of ApoE gene polymorphism with IS in the Guangxi Han populations. Furthermore, based on the data from our case-control study and previous published studies in Chinese populations, we performed a meta-analysis to investigate whether the ApoE gene polymorphism is associated with IS in Chinese populations.

## Materials and methods

### Association of ApoE gene polymorphism with IS risk in the Guangxi Han populations

#### Subject recruitment

Our hospital-based case-control study involved a total of 166 IS cases and 192 healthy controls. They were recruited from the neurology inpatient department of the First Affiliated Hospital of Guangxi University of Chinese Medicine in China from July 2009 to June 2011. All the cases were diagnosed with IS according to the criteria of the Chinese Medical Association in 1995 and the criteria amended in the fourth national cerebrovascular disease conference. Diagnoses were further confirmed by computerized tomography (CT) or magnetic resonance imaging (MRI) and the consensus of at least two experienced neurologists. Healthy controls were recruited from geriatric health checkup and volunteers of the hospital involving a total of 192 participants. We used the self-designed uniform questionnaire to record the information of all the participants. The contents of the questionnaire included the basic information, past medical history, smoking and drinking histories. The exclusion criteria of the controls were a history of stroke, brain aneurysms, Alzheimer’s disease, dementia or Parkinson’s disease. All subjects were self-reported Guangxi Han populations and unrelated to each other. Written informed consent was obtained from all study subjects and this study was approved by the ethics committees of the first affiliated hospital of Guangxi University of Chinese Medicine.

#### Blood sample collection

A peripheral blood sample (5 ml) was collected from all participants in the morning. Each sample was divided into two tubes (with or without an anticoagulant). The anticoagulation blood samples were stored in a −4°C freezer and genomic DNA was extracted within 3 days. The biochemical indicators of non-anticoagulation blood samples were detected using an automatic microplate reader.

#### Genotyping

Genomic DNA was extracted from the blood plasma using a TIANamp blood DNA kit (Catalog Number: DP318DNA; Tiangen Biotech (Beijing) Co., Ltd., Beijing, China), aliquoted into 3 tubes and stored in a −20°C freezer. Genotypes of ApoE polymorphisms were amplified by polymerase chain reaction (PCR), using the forward primer 5′-AGGGCGCTGATGGACGAGAC-3′ and the reverse primer 5′-CTCGCGGATGGCGCTGAG-3′. The amplification reaction mixture had a total volume of 20 *μ*l, which contained 10 *μ*l Taq Master mix, 0.6 *μ*l of each primer, 1 *μ*l DNA template with a density of 200–400 ng/*μ*l and a final volume of double distilled water. Each reaction mixture was initially denatured at 94°C (3 min), then underwent 35 cycles of denaturation at 94°C (30 sec), amplification by primer annealing at 62°C (30 sec) and extension at 72°C (30 sec), followed by a final extension at 72°C (5 min). The sequencing of 357bp PCR products were conducted by Beijing Sunbiotech Co., Ltd. (Beijing, China). using the ABI 3730xl DNA analyzer. The Chromas software version 2.33 was used to observe the sequencing results. Analysis of the effect of polymorphisms on the sequence of sample DNA was performed using the software ClustalX 1.81.

#### Statistical analysis

SPSS version 16.0 (SPSS Inc., Chicago, IL, USA) was used for statistical analyses. P<0.05 was considered to indicate a statistically significant difference. The Hardy-Weinberg equilibrium (HWE) test was used for the distribution of the sample and genomic frequency was assessed by the Chi-square test. Comparison between groups of enumeration data and measurement data were performed by the Chi-square test and variance analysis, respectively. Logistic regression models were used to estimate odds ratio (OR) and 95% confidence intervals (CIs) for IS risk and genotype and allele of the ApoE gene.

### Meta-analysis of the association between ApoE gene polymorphism and IS in Chinese populations

#### Literature search

Electronic databases including PubMed, Embase, Chinese Biological Medical Literature database (CBM), Chinese National Knowledge Infrastructure database (CNKI), Chinese Wanfang and Chongqing VIP database were searched from the established date to August 2012, using the search terms ‘apolipoprotein E’, ‘ApoE gene’, ‘polymorphism’, ‘ischemic stroke’, ‘cerebral infarction’, ‘brain infarction’ and ‘cerebrovascular accident’. In addition, reference lists were also examined to acquire additional relevant articles.

#### Selection criteria

All included studies had to meet the following inclusion criteria: i) published case-control studies focusing on the association between the ApoE gene and IS among all the nationalities in China; ii) studies with full text written in Chinese or English and iii) studies that follow the HWE. Exclusion criteria: i) studies that did not provide sufficient genotype and allele frequency data to assess an odds ratio (OR) with 95% confidence interval (CI) and ii) duplicate publications.

#### Data extraction

Data extraction was performed independently by two investigators. If there was any disagreement, it was resolved by consensus between the authors. A standardized, structured form was used for recording the extracted information of all relevant studies that mainly included the following: first author’s name, year of publication, country, ethnicity, number of cases and controls, source of the controls, genotyping method and distribution of genotypes and alleles. As to the distribution of genotypes and alleles, some of the studies did not provide complete genotype data, such as the allele frequencies; therefore, we had to calculate the other genotypes through the available information.

#### Statistical analysis

Statistical analysis was conducted using the Stata software version 11.1. The HWE was evaluated by the Chi-square test. In comparison with other genes, the ApoE gene has three major alleles with six possible genotypes (ɛ2/ɛ2, ɛ2/ɛ3, ɛ2/ɛ4, ɛ3/ɛ3, ɛ3/ɛ4 and ɛ4/ɛ4). Genotype ɛ3/ɛ3 was chosen as the reference category in our study. Therefore, seven genetic models were obtained (ɛ2/ɛ2 vs. ɛ3/ɛ3, ɛ2/ɛ3 vs. ɛ3/ɛ3, ɛ2/ɛ4 vs. ɛ3/ɛ3, ɛ3/ɛ4 vs. ɛ3/ɛ3, ɛ4/ɛ4 vs. ɛ3/ɛ3, allele ɛ2 vs. allele ɛ3 and allele ɛ4 vs. allele ɛ3). We assessed the strength of association between ApoE gene polymorphism and IS by odds ratio (OR) along with the corresponding 95% confidence intervals (CIs). Heterogeneity between studies was measured using the I^2^ statistic. Random effects model was used if significant heterogeneity (I^2^>50%, P<0.1) was observed between studies. Otherwise, a fixed effects model was adopted. The funnel plots and Egger’s regression test were used to examine the publication bias.

## Results

### 

#### Association of ApoE gene polymorphism with IS in the Guangxi Han populations

The genotype frequency distribution of the ApoE gene is shown in Table I. No evidence was found for a significant association between ApoE gene polymorphism and IS in the Guangxi Han populations (ɛ2/ɛ2 vs. ɛ3/ɛ3 OR=1.25, 95% CI=0.08–20.17; ɛ2/ɛ3 vs. ɛ3/ɛ3 OR=1.49, 95% CI=0.79–2.79; ɛ2/ɛ4 vs. ɛ3/ɛ3 OR=1.25, 95% CI=0.17–9.00; ɛ3/ɛ4 vs. ɛ3/ɛ3 OR=1.10, 95% CI=0.60–2.04; ɛ4/ɛ4 vs. ɛ3/ɛ3 OR=2.50, 95% CI=0.22–27.87; allele ɛ2 vs. allele ɛ3 OR=1.39, 95% CI=0.80–2.44; allele ɛ4 vs. allele ɛ3 OR=1.16, 95% CI=0.68–1.98).

### Meta-analysis of the association between ApoE gene polymorphism and IS in Chinese populations

#### Search results

The literature research through PubMed (n=28), Embase (n=42), CBM Database (n=87), CNKI database (n=136), Chinese Wan Fang (n=43) and Chongqing VIP database (n=40) yielded 376 initial publications. We screened the initial publications according to the inclusion criteria and obtained 68 studies. Among those studies, 8 studies were duplicate publications, 4 studies were published in abstract, 13 studies did not follow the HWE and 7 studies did not provide sufficient information of genotype frequency. Therefore, 36 studies were included (14–49) plus our study, making a total of 37 studies for meta-analysis.

### The characteristics of included studies

Thirty-seven studies concerning the ApoE gene polymorphism and IS were included, involving a total of 3,814 IS cases and 3,425 controls. All included studies were conducted from 1997 to 2011 in China (34 in mainland China, 2 in Hong Kong, 1 in Taiwan). There were 32 hospital-based control studies, 2 hospital and population-based control studies and 2 population-based control studies. One study did not provide information about the source of controls. The genotypes of the ApoE gene polymorphisms were investigated using PCR in all the included studies. The distribution of the ApoE genotype and allele between cases and controls and P-value for HWE of each included study is shown in Table II.

### Results of the meta-analysis

A significant association of ApoE polymorphism with IS was observed in the genetic model of ɛ2/ɛ4 vs. ɛ3/ɛ3 (OR=2.04, 95% CI=1.45–2.85), ɛ3/ɛ4 vs. ɛ3/ɛ3 (OR=1.93, 95% CI=1.42–2.62) and ɛ4/ɛ4 vs. ɛ3/ɛ3 (OR=3.41, 95% CI=2.17–5.34). However, compared with genotype ɛ3/ɛ3, no significant associations were found in the ɛ2/ɛ2 (OR=1.56, 95% CI=0.90–2.71) and ɛ2/ɛ3 genotypes (OR=0.93, 95% CI=0.79–1.09). There was no significant association between ApoE polymorphism and IS in the allele ɛ2 vs. allele ɛ3 (OR=1.10, 95% CI=0.97–1.25), while a significant association was found in allele ɛ4 vs. allele ɛ3 (OR=2.34, 95% CI=1.91–2.86).

### Publication bias

Publication bias was assessed using the funnel plots and Egger’s regression test. The genetic model of ɛ2/ɛ2 vs. ɛ3/ɛ3 (t=1.76, P=0.095), ɛ2/ɛ3 vs. ɛ3/ɛ3 (t=1.04, P=0.306), ɛ3/ɛ4 vs. ɛ3/ɛ3 (t=1.31, P=0.198), ɛ4/ɛ4 vs. ɛ3/ɛ3 (t=2.04, P=0.051) and the allelic models of ɛ2 vs. ɛ3 (t=0.13, P=0.0.897) and ɛ4 vs. ɛ3 (t=0.17, P=0.864) revealed no publication bias among the studies. However, publication bias was found in the genetic model of ɛ2/ɛ4 vs. ɛ3/ɛ3 (t=5.12, P=0.000).

## Discussion

We carried out a case-control study investigating the association between the ApoE gene and IS in Guangxi Han populations. The genotype frequencies of the ApoE gene in the control group followed HWE (P=0.994), which indicates that all the samples were suitable for genetic analysis. It is shown in Table II that no statistical significance was found between genotype and allele frequencies of ApoE and IS, which indicates that there was no significant association between ApoE gene polymorphism and IS in Guangxi Han populations. Our results were similar to a number of studies (1,13); however, they were inconsistent with another study (50). The inconsistencies may be explained by the varying distribution of the ApoE gene among different ethnicities and the different sample sizes included in the studies.

Meta-analysis collects the pooling of data from the individual inconclusive studies with a greater statistical power (51,52). Therefore, we performed a meta-analysis to investigate whether ApoE gene polymorphism is associated with IS in Chinese populations. This meta-analysis of 37 studies involved a total of 3,814 IS cases and 3,425 controls. Our meta-analysis results revealed that individuals with ɛ2/ɛ4, ɛ3/ɛ4 and ɛ4/ɛ4 genotypes were 2.04, 1.93 and 3.41 times more likely to develop IS than the individuals with the ɛ3/ɛ3 genotype, respectively. Moreover, the individuals with allele ɛ4 had a higher risk of developing IS than those with allele ɛ3 (OR=2.34, 95% CI=1.91–2.86). However, no significant differences were found in the genotype ɛ2/ɛ2 (OR=1.56, 95% CI=0.90–2.71) and ɛ2/ɛ3 (OR=0.93, 95% CI=0.79–1.09) and allele ɛ2. Therefore, the allele ɛ4 of ApoE possibly has a genetic predisposition factor for IS, which is consistent with the findings of other studies (52–54). However, our results were different to the findings in other studies (55,56), which may be due to the ethnic differences in different areas.

Heterogeneity did not exist in the genetic model of ɛ2/ɛ2 vs. ɛ3/ɛ3, ɛ2/ɛ3 vs. ɛ3/ɛ3, ɛ2/ɛ4 vs. ɛ3/ɛ3, ɛ4/ɛ4 vs. ɛ3/ɛ3 and ɛ2 vs. ɛ3; however, it did exist in the genetic models of ɛ4 vs. ɛ3 and ɛ3/ɛ4 vs. ɛ3/ɛ3. This heterogeneity may be due to the sample sizes, case diagnosis and selection, genotyping method or other risk factors. The funnel plots and Egger’s regression test revealed no significant publication bias in the majority of the genetic models, except the model of ɛ2/ɛ4 vs. ɛ3/ɛ3. One possible explanation for this is that the negative results of some studies were not published so we could not obtain this information for meta-analysis.

Several limitations must be considered in our study and the meta-analysis. Firstly, our study only involved a total of 166 cases and 192 controls and the negative association of ApoE gene polymorphism with IS may be a result of the small sample size. Secondly, the sample sizes, case diagnosis and selection, genotyping method and other risk factors may contribute to the heterogeneity in our study. Thirdly, the different sample sizes in the individual study may affect the meta-analysis results.

In conclusion, no association was found between ApoE polymorphism and IS in our case-control study, while the meta-analysis result indicates that the ApoE mutation allele ɛ4 possibly increases the risk of developing IS in Chinese populations. At present, more and more studies focus on the association between ApoE gene polymorphism and IS. However, their results are contrasting. Thus, studies with a larger sample size are needed to further confirm the association between ApoE gene polymorphism and IS.

## Figures and Tables

**Table I.** Distribution of ApoE genotypes and alleles between cases and controls of our study.

	ɛ2/ɛ2	ɛ2/ɛ3	ɛ2/ɛ4	ɛ3/ɛ3	ɛ3/ɛ4	ɛ4/ɛ4	ɛ2	ɛ3	ɛ4
IS	1	25	2	113	23	2	2	274	29
Controls	1	21	2	141	26	1	25	329	30

ApoE, apolipoprotein E; IS, ischemic stroke.

**Table II.** Characteristics and distributions of ApoE genotype and allele between cases and controls of studies included in this meta-analysis and our study.

		Sample size			Case									Control									
Study	Country	Cases	Control	Source of controls	ɛ2/ɛ2	ɛ2/ɛ3	ɛ2/ɛ4	ɛ3/ɛ3	ɛ3/ɛ4	ɛ4/ɛ4	ɛ2	ɛ3	ɛ4	ɛ2/ɛ2	ɛ2/ɛ3	ɛ2/ɛ4	ɛ3/ɛ3	ɛ3/ɛ4	ɛ4/ɛ4	ɛ2	ɛ3	ɛ4	P-value for HWE
Wang *et al*(1997)	China	40	40	Not mentioned	0	5	5	12	15	3	10	44	26	0	4	0	28	8	0	4	68	8	0.741
Yan *et al*(1997)	China	50	113	Population	0	8	1	25	15	1	9	73	18	0	19	2	78	13	1	21	188	17	0.678
Zhou *et al*(1997)	China	24	24	Hospital	0	3	3	12	4	2	6	31	11	0	2	1	19	2	0	3	42	3	0.258
Zhu *et al*(1997)	China	68	102	Hospital	1	12	0	48	6	1	14	114	8	1	9	0	80	11	1	11	180	13	0.352
Zhao *et al*(1998)	China	55	85	Hospital	0	2	0	47	6	0	2	102	6	0	11	0	68	6	0	11	153	6	0.789
Cao *et al*(1999)	China	55	85	Hospital	0	2	0	47	6	0	2	102	6	0	11	0	68	6	0	11	153	6	0.789
Peng *et al*(1999)	China	90	90	Hospital and community	0	13	1	55	19	2	14	142	24	1	16	1	63	8	1	19	150	11	0.685
Ding *et al*(2000)	China	58	46	Hospital	1	3	2	37	13	2	7	90	19	0	2	1	38	5	0	3	83	6	0.286
Wang *et al*(2000)	China	50	50	Hospital	0	6	1	36	7	0	7	85	8	0	7	2	34	7	0	9	82	9	0.457
Yu *et al*(2000)	China	63	30	Hospital	0	4	1	36	18	4	5	94	27	0	3	0	23	4	0	3	53	4	0.914
Li *et al*(2001a)	China	63	66	Hospital	0	9	0	37	14	3	9	97	20	0	10	2	49	5	0	12	113	7	0.280
Li *et al*(2001b)	China	43	60	Hospital	0	5	1	22	13	2	6	62	18	0	9	2	41	8	0	11	99	10	0.530
Zhang *et al*(2001a)	China	75	40	Hospital	0	6	3	40	22	4	9	108	33	0	8	1	28	3	0	9	67	4	0.720
Zhang *et al*(2001b)	China	116	40	Hospital	0	9	6	65	30	6	15	169	48	0	8	1	28	3	0	9	67	4	0.720
Zhu *et al*(2002)	China	49	108	Hospital	0	7	1	23	16	2	8	69	21	1	14	3	72	17	1	19	175	22	0.847
Wen *et al*(2002a)	China (HK)	67	134	Hospital and community	4	7	2	41	11	2	17	100	17	2	24	3	89	15	1	31	217	20	0.925
Wen *et al*(2002b)	China	673	88	Hospital	10	67	99	234	227	36	186	762	398	1	9	7	50	18	3	18	127	31	0.068
Shen *et al*(2003)	China	66	90	Hospital	0	7	4	33	20	2	11	93	28	0	12	2	68	8	0	14	156	10	0.423
Wang *et al*(2003)	China	40	60	Population	1	1	2	20	16	0	5	57	18	0	3	1	43	13	0	4	102	14	0.650
Jin *et al*(2004)	China (Taiwan)	226	201	Hospital	2	14	3	152	52	3	21	370	61	2	17	2	156	22	2	23	351	28	0.197
Xiao *et al*(2005)	China	254	211	Hospital	0	26	2	191	33	2	28	441	39	3	29	3	143	29	4	38	344	40	0.263
Baum *et al*(2006)	China (HK)	243	311	Hospital	7	39	6	155	32	4	59	381	46	2	60	6	203	39	1	70	505	47	0.659
Gao *et al*(2006)	China	100	100	Hospital	1	11	0	75	13	0	13	174	13	1	13	0	80	6	0	15	179	6	0.809
Li *et al*(2006)	China	51	69	Hospital	1	2	3	26	19	0	7	73	22	0	4	2	53	10	0	6	120	12	0.160
Ma *et al*(2006)	China	109	50	Hospital	3	10	2	61	27	6	18	159	41	0	6	1	38	5	0	7	87	6	0.743
Wang *et al*(2006)	China	115	120	Hospital	1	8	3	85	16	2	13	194	23	2	15	2	92	9	0	21	208	11	0.352
Xie *et al*(2006)	China	45	100	Hospital	1	4	0	28	11	1	6	71	13	0	7	1	86	6	0	8	185	7	0.534
Zhao *et al*(2006)	China	13	116	Hospital	0	1	0	11	1	0	1	24	1	0	15	1	90	9	1	16	204	12	0.492
Zhou *et al*(2006)	China	72	68	Hospital	2	11	2	52	5	0	17	120	7	2	9	0	46	11	0	13	112	11	0.155
He *et al*(2007)	China	108	90	Hospital	0	17	4	61	25	1	21	164	31	0	10	2	71	7	0	12	159	9	0.256
Man *et al*(2007)	China	40	50	Hospital	1	6	0	20	10	3	8	56	16	1	6	0	38	4	1	8	86	6	0.109
Lu *et al*(2008)	China	115	120	Hospital	1	8	3	85	16	2	13	194	23	1	8	3	89	17	2	13	203	24	0.144
Sun *et al*(2008)	China	78	90	Hospital	0	12	3	44	18	1	15	118	23	0	10	2	71	7	0	12	159	9	0.256
Xie *et al*(2008)	China	184	50	Hospital	0	18	8	98	56	4	26	270	72	1	10	2	36	1	0	14	83	3	0.066
Wang *et al*(2009)	China	92	86	Hospital	3	10	2	39	28	10	18	116	50	0	9	2	65	9	1	11	148	13	0.392
Xu *et al*(2010)	China	58	50	Hospital	0	4	2	28	23	1	6	83	27	0	3	2	39	6	0	5	87	8	0.059
Gu *et al*(2011)	China	166	192	Hospital	1	25	2	113	23	2	29	274	29	1	21	2	141	26	1	25	329	30	0.994

The ethnicity of all subjects was Asian. The genotyping method was polymerase chain reaction in all studies. HWE, Hardy-Weinburg equilibrium; ApoE, apolipoprotein E.
